# Ants of the *Monomorium monomorium* species-group (Hymenoptera: Formicidae) in the Arabian Peninsula with description of a new species from southwestern Saudi Arabia

**DOI:** 10.7717/peerj.4277

**Published:** 2018-02-01

**Authors:** Mostafa Sharaf, Hathal M. Al Dhafer, Abdulrahman S. Aldawood, Francisco Hita Garcia

**Affiliations:** 1 Department of Plant Protection, College of Food and Agriculture Sciences, King Saud University, Riyadh, Kingdom of Saudi Arabia; 2 Division of Invertebrate Zoology, American Museum of Natural History, New York, NY, USA; 3 Okinawa Institute of Science and Technology, Okinawa, Japan

**Keywords:** Middle East, Asir Province, Revision, Myrmicinae, Palearctic region, Identification key

## Abstract

We revise the taxonomy of the myrmicine ants of the *Monomorium monomorium* species-group for the Arabian Peninsula. Six species are recognized: *Monomorium aeyade* Collingwood & Agosti, 1996, *M. clavicorne* André, 1881, *M. exiguum* Forel, 1894, *M*. *holothir* Bolton, 1987, *M. mohammedi* sp. n., and *M. sarawatense* Sharaf & Aldawood, 2013. On the basis of the worker caste, we describe *Monomorium mohammedi* sp. n. from the southwestern region of the Kingdom of Saudi Arabia (KSA). We designate a neotype for *Monomorium aeyade* Collingwood & Agosti and redescribe and illustrate the worker caste. Furthermore, we provide a worker-based species identification key, distribution maps for the treated species, and ecological and biological notes, if available. *Monomorium holothir* is recorded for the first time from the KSA. Also, we propose *M. clavicorne* var. *punica* Santschi, 1915a as a junior synonym of *M. clavicorne*, as well as *M. dryhimi* Aldawood & Sharaf, 2011 and *M. montanum* Collingwood & Agosti, 1996 to be treated as junior synonyms of *Monomorium exiguum*.

## Introduction

The ant genus *Monomorium* is one of the most diverse genera in the subfamily Myrmicinae, with 388 described species and subspecies (Bolton, 2017). It is distributed worldwide throughout all zoogeographic regions, with most species occurring in the Old World tropics and temperate zones (Brown, 2000). Considering how diverse and widespread the genus is, very little information on the natural history of most species exists, especially for the *M. monomorium* species-group (Bolton, 1987). Apparently, most species inhabit the topsoil layer or leaf litter and seem to have a rather generalist diet. The taxonomic foundation for the genus as a whole is in a moderate state based on some regional revisions (e.g., Bolton, 1987; Heterick, 2001; Heterick, 2006), as well as faunistic treatments providing local or regional keys (e.g., Collingwood & Agosti, 1996; Terayama, 2009; Sarnat & Economo, 2012). The available revisionary contributions on the fauna of the *M. monomorium* species-group are scarce, and basically consist of Bolton (1987) for the Afrotropical fauna and Heterick (2001) and Heterick (2006) for the Australian and Malagasy faunas respectively. However, these include many species now removed from the genus.

The taxonomic history of the *M. monomorium* species-group in the Arabian Peninsula is a set of single distribution records and single species descriptions scattered through the literature. The first published work on the Arabian *Monomorium* fauna (Collingwood, 1985) recorded a couple of species of the *M. monomorium* species-group from the Kingdom of Saudi Arabia (KSA), namely *M. clavicorne* André, 1881 and *M. montanum* Collingwood & Agosti, 1996 (initially misidentified as *M. zulu* Santschi, 1914 in Collingwood, 1985). In a later faunistic contribution Collingwood & Agosti (1996) listed eight species, and described five new species, *M. aeyade* Collingwood & Agosti, 1996, *M. baushare* Collingwood & Agosti, 1996, *M. desertorum* Collingwood & Agosti, 1996, *M. montanum* Collingwood & Agosti, 1996, and *M. qarahe* Collingwood & Agosti, 1996.

The first record of the species *M. exiguum* Forel was published by Aldawood & Sharaf (2009) from the Asir Mountains (KSA). Aldawood & Sharaf (2011) described *M. dryhimi*, based on the worker caste from the southwestern Mountains of the KSA, and provided a key to the Arabian species. El-Hawagryi et al. (2013) described *M. sarawatensis* Sharaf & Aldawood from the Al Baha Province based on the worker caste, and provided a key to the Arabian species of the *M. monomorium* species-group. Recently, *M. desertorum* Collingwood & Agosti, 1996 was synonymized with *M. exiguum* Forel, 1894 (Sharaf et al., 2015), and Sharaf et al. (2017) produced a key to the *Monomorium* fauna of the Socotra Archipelago and described *M. elghazalyi* Sharaf & Aldawood, 2017, and synonymized *M. baushare* and *M. qarahe* under *M. exiguum*.

In the Malagasy region, *M. exiguum* is the most abundant species in leaf litter samples (Heterick, 2006). However, this group of ants is taxonomically difficult due to lack of revisionary work, in addition to the small body size and pale body colors that make the ants frequently overlooked by collectors and consequently poorly represented in the regional museums and collections. Ants of the *M. monomorium*-group are among the most abundant *Monomorium* in the Arabian Peninsula, commonly represented in ecological and biodiversity research projects of the region.

In this study, we provide the first comprehensive revision of the *M. monomorium* species-group for the Arabian Peninsula. We describe one new species from the southwestern region of the KSA and re-describe the five previously known species. For all species we present detailed descriptions, diagnoses, high-quality montage images, and distribution maps. Furthermore, we provide a new illustrated identification key to the members of the species group on the basis of the worker caste.

## Material and Methods

**Figure 1:**
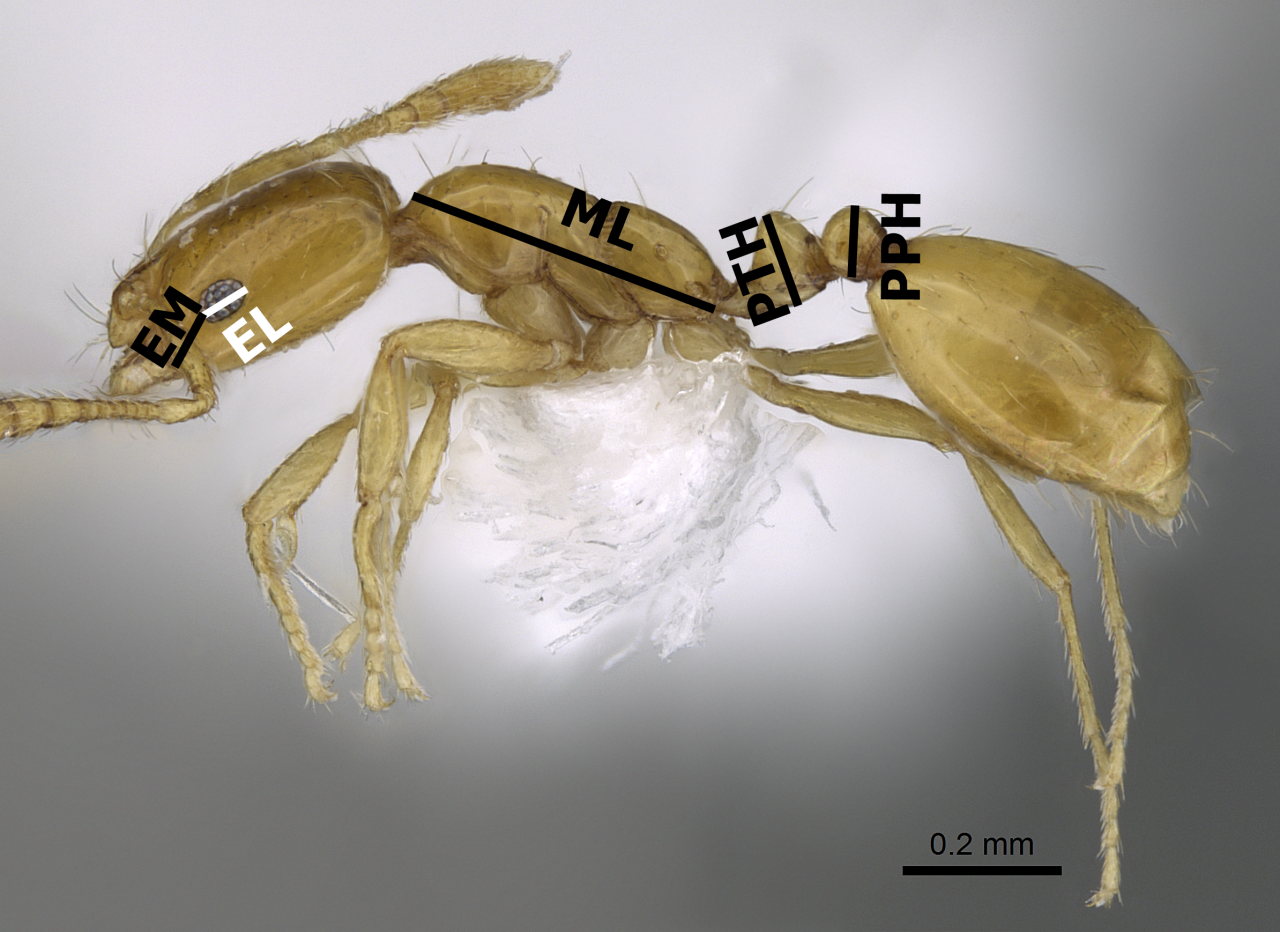
Body profile of *M. exiguum Forel* (CASENT0922302, from https://www.antweb.org/specimenImages.do?name=casent0922302countryName=South%20Africa—Michele Esposito) illustrating the used measurements.

**Figure 2:**
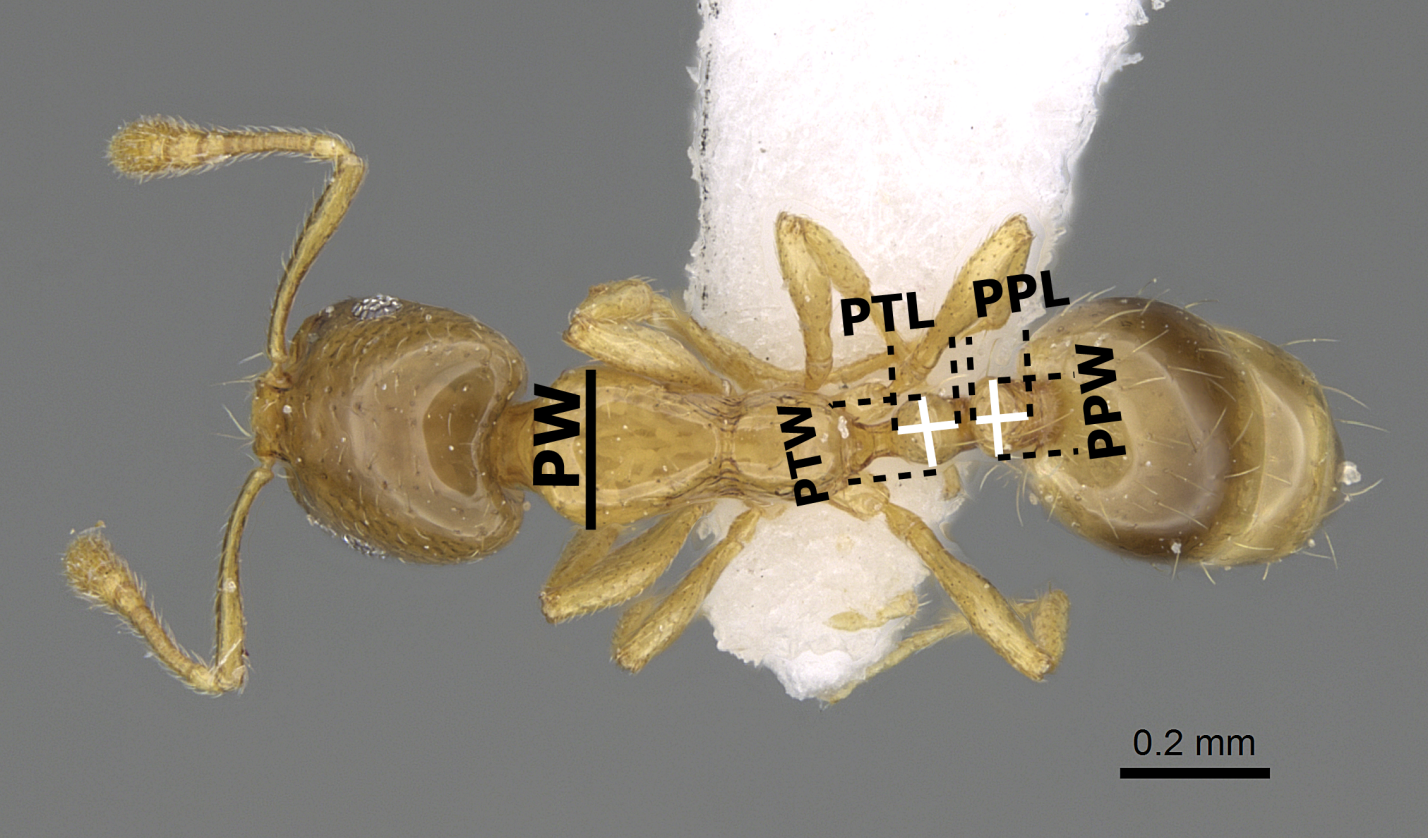
Body in dorsal view of *M. exiguum* Forel (CASENT0922344, from https://www.antweb.org/specimen.do?name=casent0922344—Michele Esposito) illustrating the used measurements.

**Figure 3:**
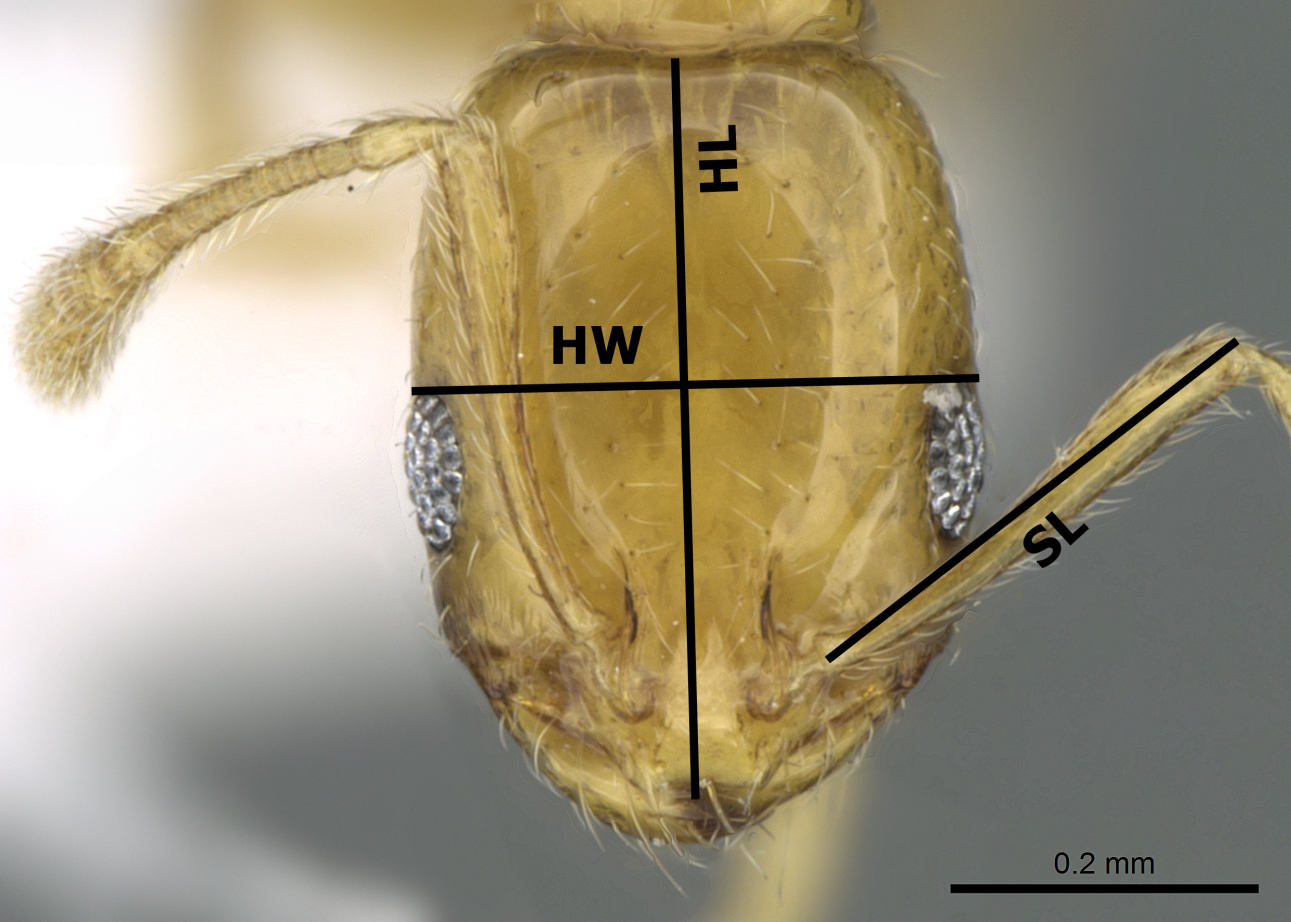
Head in full-face view of *M. holothir* Bolton (CASENT0906392, from https://www.antweb.org/specimenImages.do?name=casent0906392countryName=Seychelles—Estella Ortega) illustrating the used measurements.

The species names follow the online catalogue of ants of the world (Bolton, 2017). Distribution maps were made using DIVA-GIS (version 7.5.0.0). Digital color images of lateral and dorsal views of the entire body and full-face views of the head of each species were created using a Leica DFC450 digital camera with a Leica Z16 APO microscope and LAS (v3.8) software. These images are also available online on AntWeb (http://www.AntWeb.org) and are accessible through unique specimen identifiers attached to each pin (e.g., CASENT0922329). Throughout the text, ‘w’ stands for ‘worker’ or ‘workers’. Morphometric measurements are shown in Figs. 1–3.

### Nomenclatural acts

The electronic version of this article in Portable Document Format (PDF) will represent a published work according to the International Commission on Zoological Nomenclature (ICZN), and hence the new names contained in the electronic version are effectively published under that Code from the electronic edition alone. This published work and the nomenclatural acts it contains have been registered in ZooBank, the online registration system for the ICZN. The ZooBank LSIDs (Life Science Identifiers) can be resolved and the associated information viewed through any standard web browser by appending the LSID to the prefix http://zoobank.org/. The LSID for this publication is: urn:lsid:zoobank.org:pub:58913E59-AEE4-43BD-881E-C23CD61F44F9. The online version of this work is archived and available from the following digital repositories: PeerJ, PubMed Central and CLOCKSS.

## Results

### Diagnosis of Arabian ants in the *Monomorium monomorium* species-group

Within the genus *Monomorium*, workers of the *M. monomorium* species-group can be easily recognized by the following combination of characters (Bolton, 1987): monomorphic, with size variation; median clypeal portion raised, projecting anteriorly and longitudinally bicarinate; anterior clypeal margin without a pair of teeth; dorsal surface of mandibles unsculptured and masticatory margin armed with four teeth, decreasing in size from apex to base; antennae with 10–12 segments, terminating in a well-defined three-segmented club; eyes present but variable in size, situated in front of the midlength of the sides in full-face view, and with four or more ommatidia in the longest row; head longer than broad; cephalic dorsum smooth and shining; metanotal groove impressed, with distinct cross-ribs; propodeal spiracle circular to subcircular; propodeal dorsum meeting declivity in a rounded angle; promesonotum and propodeal dorsum unsculptured; body pilosity variable in distribution but usually conspicuous, rarely absent from mesosomal dorsum; petiole, postpetiole and gastral tergites usually unsculptured.

### Synoptic species list of Arabian ants in the *Monomorium monomorium* species-group

**Table:** 

*Monomorium aeyade* Collingwood & Agosti, 1996
*Monomorium clavicorne* André, 1881
= *Monomorium clavicorne punicum* Santschi, 1915a; **syn. nov.**
*Monomorium exiguum* Forel, 1894
= *Monomorium exiguum* var. *bulawayensis* Forel, 1913b
= *Monomorium faurei* Santschi, 1915b
= *Monomorium exiguum* r. *flavescens* Forel, 1916
= *Monomorium montanum* Collingwood & Agosti, 1996 **syn. nov.**
= *Monomorium dryhimi* Aldawood & Sharaf, 2011 **syn. nov.**
*Monomorium* *holothir* Bolton, 1987
*Monomorium mohammedi* Sharaf & Hita Garcia **sp. n.**
*Monomorium sarawatense* Sharaf & Aldawood, 2013

### Identification key to the Arabian species of the *Monomorium monomorium*-group

**Table:** 

1.	Antenna 12-segmented	2
	–Antenna 11-segmented	3
2.	Body pilosity clubbed; mesosoma, petiole and postpetiole distinctly sculptured (Fig. 4A)	***sarawatense*** **Sharaf & Aldawood**
	–Body pilosity simple; mesosoma, petiole and postpetiole smooth and shining (Fig. 4B)	***holothir*** **Bolton**
3.	Mesosoma without standing hairs (Figs. 4C and 4D)	4
	–Mesosoma with standing hairs (Figs. 4E and 4F)	5
4.	Eyes appearing larger, with a ring of seven to eight ommatidia encircling a single row of 2 ommatidia, and in profile closer to mandibular insertions (EM 0.05); meso-and metapleuron smooth; petiole and postpetiole smooth and each with one pair of standing hairs (Fig. 4C)	***aeyade*** **Collingwood & Agosti**
	–Eyes appearing smaller, with only 5–6 ommatidia, and in profile further away from mandibular insertions (EM 0.09–0.11); meso-and metapleuron finely shagreened; petiole and postpetiole superficially shagreened and without standing hairs (Fig. 4D)	***mohammedi*** **sp. n.**
5.	Mesosoma with only two pairs of standing hairs, one on pronotal corners and one propodeum (Fig. 4E)	***clavicorne*** **André**
	–Mesosoma with several pairs of standing hairs, about 10 pairs (Fig. 4F)	***exiguum*** **Forel**

### Review of species

**Table:** 

***Monomorium aeyade* Collingwood & Agosti, 1996, Fig. 5**
*Monomorium aeyade* Collingwood & Agosti, 1996: 341 (w.)

**Type material examined**

**Neotype** pinned worker: Oman, Wadi Aeyad, 20.iii.1990, (MD Gallagher) (WMLC: CASENT0922329).

**Measurements**

Paratype worker.

TL 1.42; HL 0.40; HW 0.31; SL 0.27; EL 0.06; ML 0.40; PW 0.18; PTW 0.07; PTH 0.12; PPW 0.09; PPH 0.09; CI 78; EI 19; SI 87.

**Figure 4:**
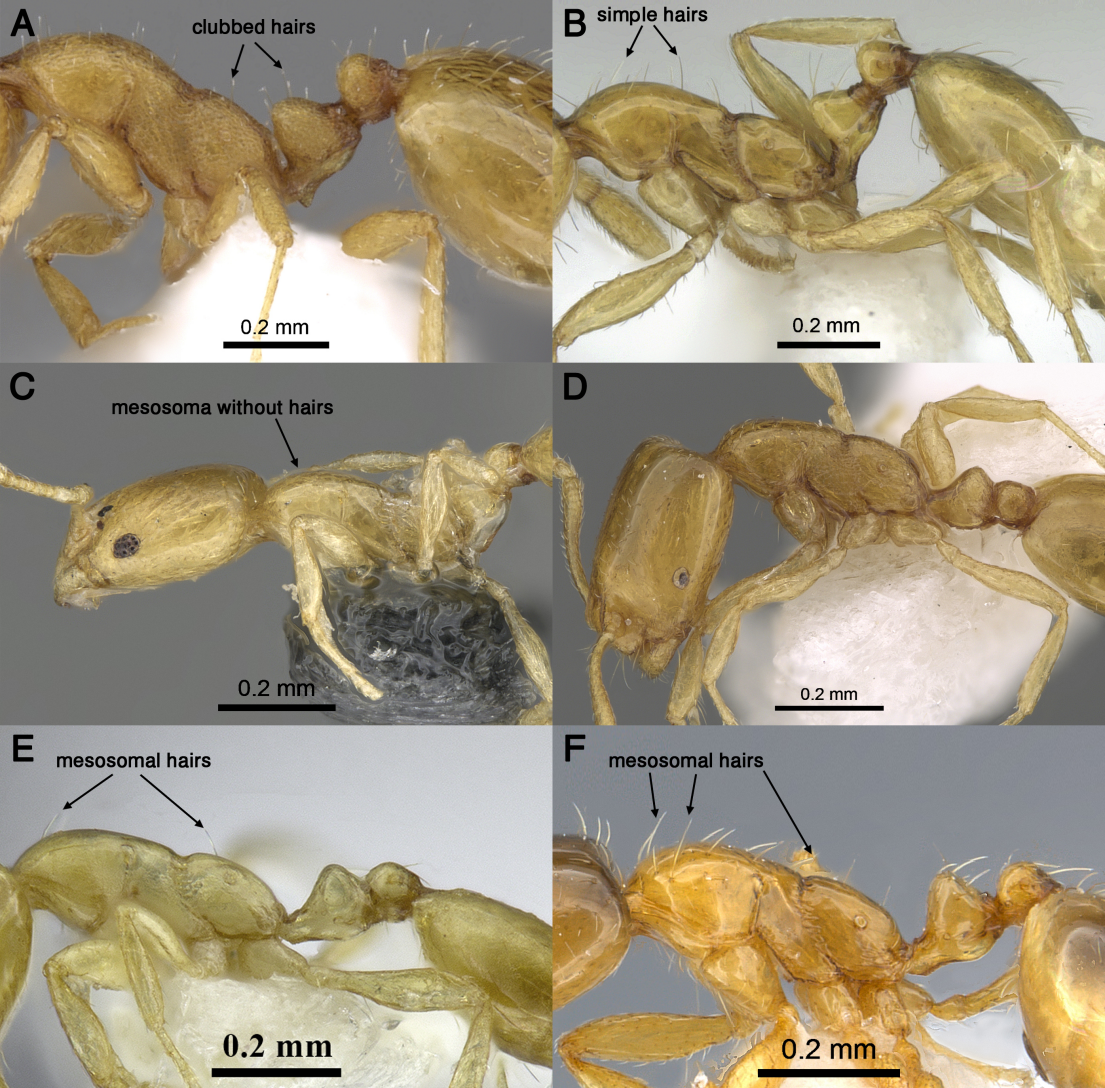
Body in profile showing eyes, pilosity and sculpture on mesosoma and waist segments. (A) *M. sarawatense* (CASENT0280971, from https://www.antweb.org/bigPicture.do?name=casent0280971&shot=d&number=1, photographer: Estella Ortega). (B) *M. holothir* (CASENT0906392, from https://www.antweb.org/specimenImages.do?name=casent0906392&countryName=Seychelles, photographer: Estella Ortega). (C) *M. aeyade* (CASENT0922329, from https://www.antweb.org/bigPicture.do?name=casent0922329&shot=l&number=1, photographer: Michele Esposito). (D) *M. mohammedi* (CASENT0922351, https://www.antweb.org/images.do?genus=monomorium&species=mohammedi&rank=species&countryName=Saudi%20Arabia, photographer: Michele Esposito). (E) *M. clavicorne* (CASENT0823774, https://www.antweb.org/specimen.do?name=casent0823774, photographer: Francisco Hita Garcia). (F) *M. exiguum* (CASENT0217367, https://www.antweb.org/specimen.do?name=casent0217367, photographer: Erin Prado).

**Figure 5:**
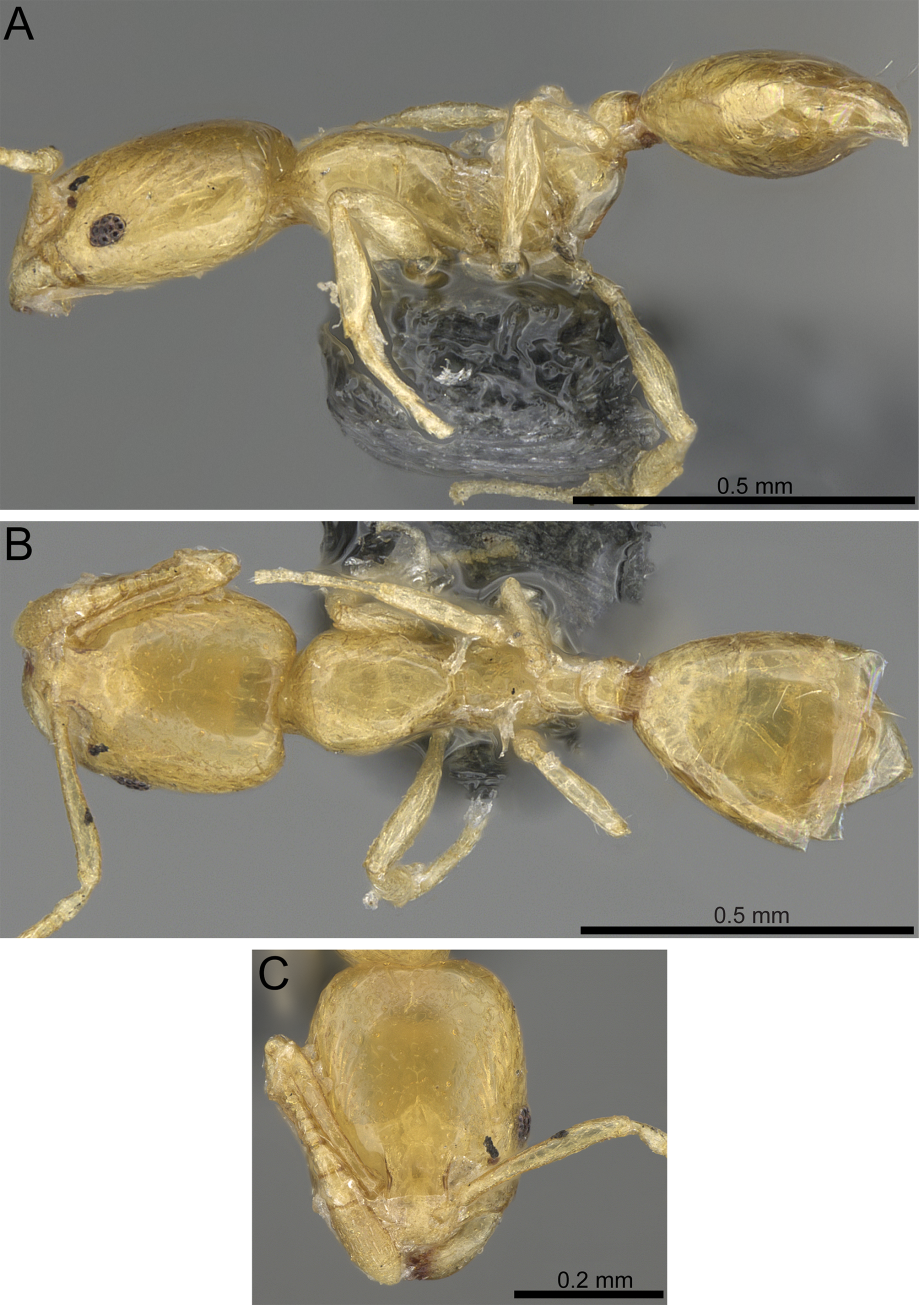
*Monomorium aeyade* (CASENT0922329, from https://www.antweb.org/specimen.do?name=casent0922329, photographer: Michele Esposito). (A) Body in profile. (B) Body in dorsal view. (C) Head in full-face view.

**Description**

**Worker. Head**. In full-face view distinctly longer than broad with nearly parallel sides and feebly concave posterior margin; median clypeal portion without carina or anterolateral angles, anterior clypeal margin straight or feebly concave; antenna 11-segmented; scapes relatively long (SI 81), when laid straight back from their insertions reach three quarter of head length; eyes oval, small, (EL 0.16 × HW) with a ring of ommatidia encircling a single row of 2 ommatidia; frontal lobes farther apart in full-face view. **Mesosoma.** In profile with flat promesonotal dorsum, which slopes posteriorly to a well-defined metanotal groove; propodeal spiracles small and pinhole-like; propodeal dorsum evenly sloping posteriorly to short declivity. **Petiole.** Node massive, rounded dorsally, and little higher than postpetiolar node in profile; anterior peduncle short. **Postpetiole**. Node low and convex dorsally. **Sculpture.** Entire body surfaces smooth and shining except for the distinct metanotal cross ribs. **Pilosity**. Underside of head without hairs; cephalic surface with scattered minute hair-pits; anterior clypeal margin and mandibles with longer hairs; antennae with abundant appressed pubescence; mesosoma without hairs; postpetiole with two pairs of backward directed hairs; gaster with few longer hairs. **Color**. Uniform light yellow.

**Table 1:** List of species with known distribution ranges. Data extracted from Antmaps (http://antmaps.org; Janicki et al., 2016).

**Species**	**Distribution**
*M. aeyade*	Oman
*M. clavicorne*	Egypt, Iran, Israel & Palestine, KSA, Lebanon, Morocco, Sudan, Syria, Tunisia, Turkey, United Arab Emirates
*M. exiguum*	Angola, Cameroon, Cape Verde, Central African Republic, Democratic Republic of Congo, Ethiopia, Gabon, Ghana, Guinea, Ivory Coast, Kenya, Iran, KSA, Madagascar, Mozambique, Nigeria, Oman, Spain, South Africa, Tanzania, Uganda, United Arab Emirates, Yemen, Zimbabwe
*M. holothir*	Kenya, KSA
*M. mohammedi*	KSA
*M. sarawatense*	KSA

**Figure 6:**
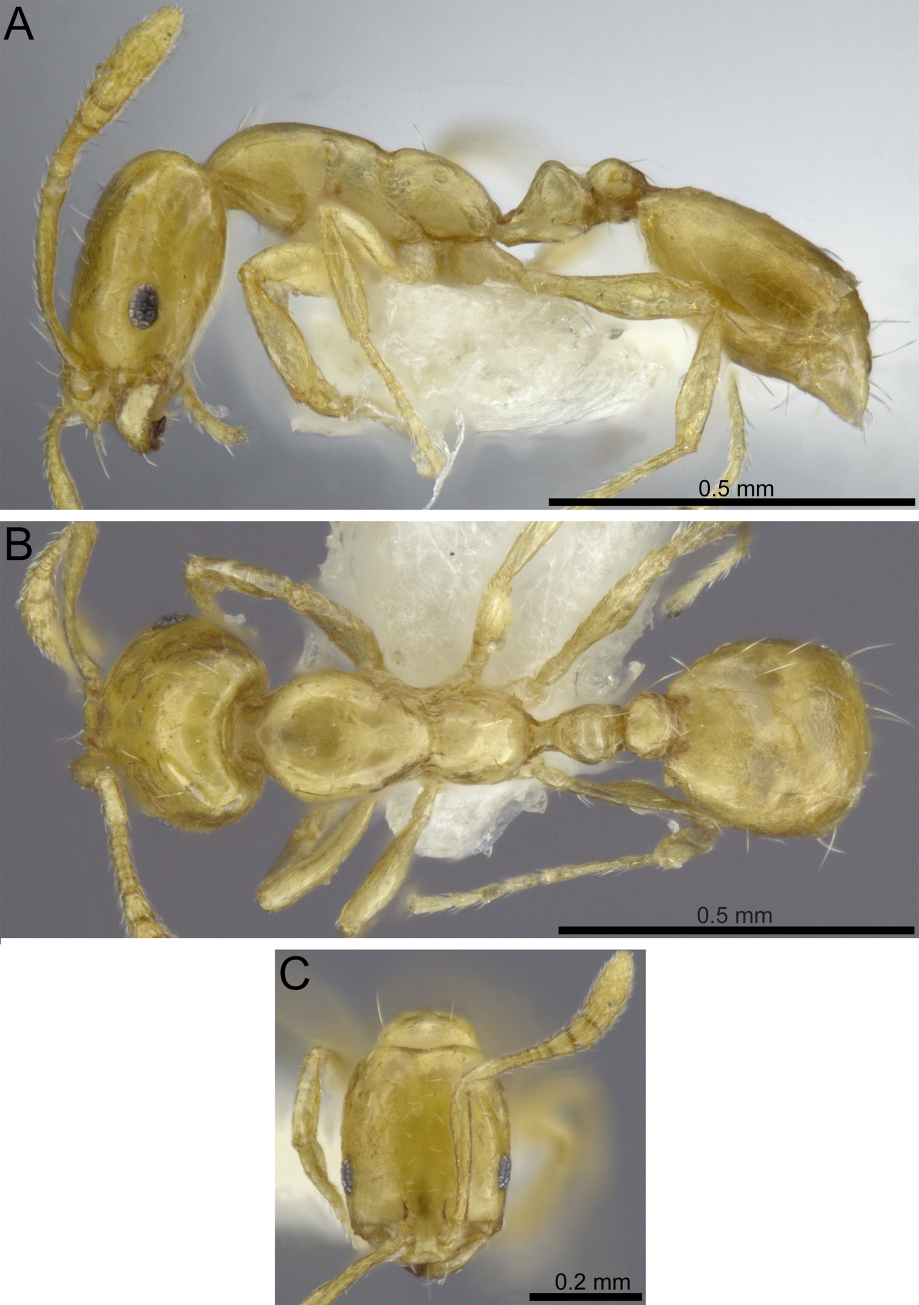
*Monomorium clavicorne* (photographer: Francisco Hita Garcia). (A) Body in profile. (B) Body in dorsal view. (C) Head in full-face view.

**Note**

This species was originally described based on two worker specimens, the holotype and one paratype. During an extensive search in the WMLC collection it was not possible to locate the holotype, which is presumably lost. However, the paratype specimen was available for examination. The original description given by Collingwood & Agosti (1996) was brief and the diagnostic differentiation was unclear, therefore, we designate a neotype to unequivocally ascertain the identity of the species and re-describe the species.

**Biological and ecological notes**

Nothing is known of the biology or ecology of the species.

**Geographic range**

*Monomorium aeyade* is only known from the type locality in Oman (Table 1).

**Table:** 

***Monomorium clavicorne* André, 1881, Fig. 6**
*Monomorium clavicorne* André, 1881: 68, pl. 3, fig. 9 (w.). [Combination in *Monomorium* (*Lampromyrmex*): Wheeler 1922: 876. Subspecies of *Monomorium orientale*: Mayr, 1904: 4; Emery, 1908: 685. Raised to species by Santschi 1915a: 58; Emery, 1922: 183. Current subspecies: nominal plus *Monomorium clavicorne punicum*].
*Monomorium clavicorne* var. *punica* Santschi, 1915a: 58, fig. 4 (w.). **Syn. nov.**

**Type material examined**

**Of** ***M. clavicorne*: Holotype**, pinned worker, **ISRAEL**, Jaffa (MNHN: CASENT0915416) [image examined].

**Of** ***M. clavicorne punicum*: Holotype**, pinned worker, **TUNISIA**: Sousse, 31.xii.1915 (Normand) (NHMB: CASENT0913568) [image examined].

[Note: Based on the examination of the type images of both, *M. clavicorne* and *M. clavicorne punicum*, we propose to synonymize the latter under the first on the basis of morphological similarity and biogeographical considerations].

**Non-type material examined**

**KSA:** Riyadh, Al Hayer, 24.394839°N, 46.832231°E, 544 m, 10.iii.2011, (Sharaf MR) (1 w, KSMA).

**Previous records.** Riyadh, Riyadh Agricultural Centre, 24.501389°N, 46.626111°E, 22.iii.83, 19.iv.83; Fayfa, 17.28797°N, 43.14434°E, 27.iii.83; Al Qatif, 26.510278°N, 49.968889°E, 15.iv.83; Hair valley, 17.iv.83 (Collingwood CA) (Collingwood, 1985).

**Measurements**

**Worker** (*n* = 2). TL 1.44–1.48; HL 0.37–0.38; HW 0.28–0.29; SL 0.22–0.25; EL 0.05–0.06; ML 0.41–0.42; PW 0.17–0.18; PTL 0.07–0.08; PTW 0.07; PTH 0.09–0.12; PPL 0.05–0.07; PPW 0.08; PPH 0.07–0.08; CI 76; EI 17–21; SI 79–86.

**Description**

**Worker. Head**. In full-face view distinctly longer than broad with nearly parallel sides and feebly concave posterior margin; median clypeal portion without carina or anterolateral angles, anterior clypeal margin feebly concave; antenna 11-segmented; terminal funicular segment enlarged, more than twice longer than the two preceding segments; scapes long (SI 79–86); eyes oval, small, (EL 0.17–0.21 × HW) with a ring of ommatidia encircling two inner short rows of 2–3 ommatidia; frontal lobes farther apart in full-face view. **Mesosoma.** In profile with a feebly convex promesonotal dorsum, which slopes posteriorly to a well-defined metanotal groove; propodeal spiracles small and pinhole-like; propodeal dorsum evenly sloping posteriorly to short declivity. **Petiole.** Node massive, narrowly rounded above, and slightly higher than postpetiolar node in profile, anterior peduncle short. **Postpetiole**. Node small and convex dorsally. **Sculpture.** Cephalic surface smooth and shining; mandibles smooth and shining, with faint striations; mesosoma, petiole, postpetiole, and gaster smooth and shining; metanotal cross ribs distinct. **Pilosity**. Cephalic surface with scattered minute hair-pits; anterior clypeal margin and mandibles with longer hairs; antennae with abundant appressed pubescence; pronotal angles with a pair of long hairs; propodeal dorsum with one pair of hairs; petiole and postpetiole each with one pair of backward directed hairs; gaster with few longer hairs on distal half. **Color**. Overall uniform clear yellow.

**Biological and Ecological notes**

Little is known of the biology of the species. The single specimen available was found in a cultivated area with a sewage water stream. It was coexisting with *Tapinoma simrothi* Krausse, 1911, *Trichomyrmex mayri* (Forel, 1902), and *Tetramorium caespitum* (Linnaeus, 1758). Due to the relatively broad regional distribution of the species it might be an introduction to the KSA.

**Geographic range**

This species was described from Palestine (André, 1881) and recorded from many countries in the Middle East including Iran, Lebanon, Sudan, Syria, Turkey, the KSA (Collingwood, 1985; Collingwood & Agosti, 1996), the UAE (Collingwood et al., 2011), and several North African countries including Egypt (Sharaf, 2006), Morocco and Tunisia (Table 1).

**Table:** 

***Monomorium exiguum* Forel, 1894, Fig. 7**
*Monomorium exiguum* Forel, 1894: 85. [Combination in *Monomorium* (*Martia*): Forel, 1913a: 351; in *Monomorium* (*Lampromyrmex*): Wheeler, 1922: 876].
*Monomorium exiguum* var. *bulawayensis* Forel, 1913: 217 (w.) [Combination in *Monomorium* (*Lampromyrmex*): Wheeler, 1922: 876. Junior synonym of *Monomorium exiguum*: Bolton, 1987: 388; here confirmed].
*Monomorium faurei* Santschi, 1915b: 260, fig. 10 (w.). [Combination in *Monomorium* (*Lampromyrmex*): Wheeler, 1922: 876. Junior synonym of *Monomorium exiguum*: Bolton, 1987: 388; here confirmed].
*Monomorium exiguum* r. *flavescens* Forel, 1916: 418 (w.). [Junior synonym of *Monomorium exiguum*: Bolton, 1987: 388; here confirmed].
*Monomorium minutissimum* Santschi, 1937: 227, figs. 27, 28 (w.). [Junior synonym of *Monomorium mictile*: Bolton, 1987: 401. Junior synonym of *Monomorium exiguum*: Heterick, 2006: 116; here confirmed].
*Monomorium baushare* Collingwood & Agosti, 1996: 342 (w.). [Junior synonym of *Monomorium exiguum*: Sharaf et al., 2017: 343; here confirmed].
*Monomorium desertorum* Collingwood & Agosti, 1996: 344 (w.). [Junior synonym of *Monomorium exiguum*: Sharaf et al., 2015: 52; here confirmed].
*Monomorium montanum* Collingwood & Agosti, 1996: 350, fig. 24 (w.). **Syn. nov.**
*Monomorium qarahe* Collingwood & Agosti, 1996: 353 (w.). [Junior synonym of *Monomorium exiguum*: Sharaf et al., 2017: 343; here confirmed].
*Monomorium dryhimi* Aldawood & Sharaf, 2011: 49, figs. 1–7 (w.). **Syn. nov.**

**Type material examined**

**Of** ***M. exiguum***: **Lectotype**, pinned worker, **ETHIOPIA**: Shoa, 3 (MHNG: CASENT0101870). **Paralectotype**, Shoa, 3 (MHNG: CASENT0101853) [Images of both examined].

**Figure 7:**
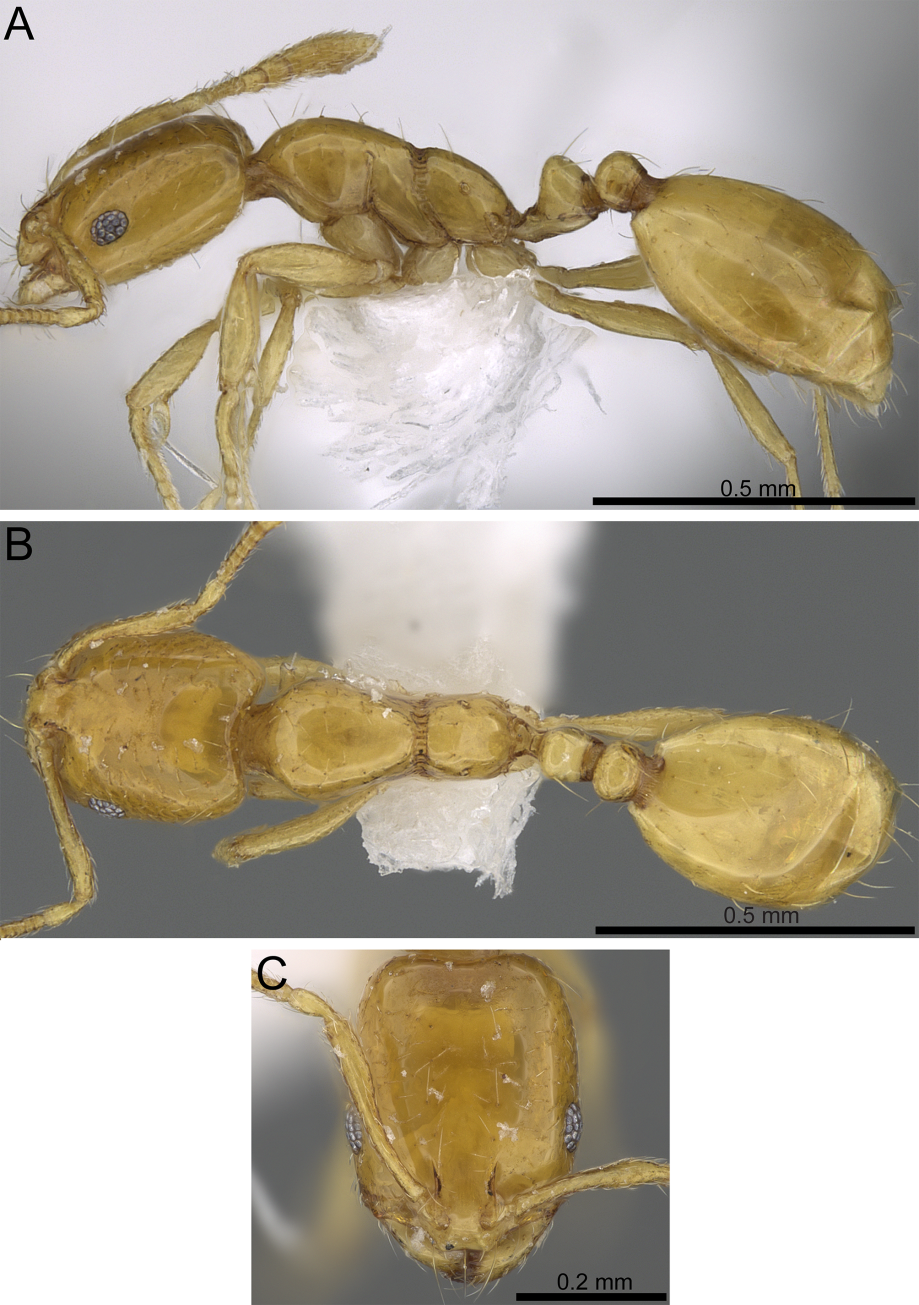
*Monomorium exiguum* (CASENT0922344, from https://www.antweb.org/specimen.do?name=casent0922344, photographer: Michele Esposito). (A) Body in profile. (B) Body in dorsal view. (C) Head in full-face view.

**Of** ***M. baushare***: **Holotype**, pinned worker, **OMAN**: Sad Baushar, 23.55, 58.4, 2.I.1992 (Gallagher, MD) (WMLC: CASENT0906342) [examined].

**Of** ***M.*** ***bulawayensis***: **Lectotype**, pinned worker, **ZIMBABWE**: Bulawayo, (Arnold, G) (MHNG: CASENT0010763). **Paralectotype**, pinned worker with same data as lectotype (MHNG: CASENT0010762) [Images of both examined].

**Of** ***M. dryhimi***: **Holotype**, pinned worker, **KSA**: Al Bahah province, Amadan forest, Al Mandaq governorate, 20.7496°N, 41.24743°E, 1,881 m, 19.v.2010 (Sharaf, MR & Aldawood, AS) (KSMA). **Paratypes**, 15 pinned workers with same data as holotype (CASC: CASENT0217367; KSMA; WMLC: CASENT0922344).

**Of** ***M.*** ***faurei***: **Lectotype**, pinned worker, **GABON**: 31.xii.1914 (Faure, F) (NHMB: CASENT0010878). **Paralectotype**, pinned worker with same data as lectotype (NHMB: CASENT0010879) [Images of both examined].

**Of** ***M. flavescens***: **Lectotype**, pinned worker, **DEMOCRATIC REPUBLIC OF CONGO**: St. Gabriel, Stanleyville (Kohl) (MHNG: CASENT0101592). **Paralectotype**, pinned worker with same data as lectotype (MHNG: CASENT0101586) [Images of both examined].

**Of** ***M.*** ***minutissimum***: **Holotype**, pinned worker, Angola, Chemin d’ Ebanga (Monard, A) (NHMN: CASENT0010880) [Images examined].

**Of** ***M. montanum***: **Holotype**, pinned worker, **KSA**: Sawdah Mt., 2500 m, 9.iv.1983 (Collingwood, CA) (WMLC). **Paratypes**, 4 pinned workers: 1 w same data as holotype (NHMB: CASENT0913825); 1 w Wadi Azizah, 18.ix.1983 (Collingwood, CA) (WMLC: CASENT0906347); 1 w Bishah, 7.iv.1983 (Collingwood, CA) (WMLC: CASENT0906345); 1 w An-Naamah, 8.iv.1983 (Collingwood, CA) (WMLC: CASENT0906346) [examined].

**Of** ***M. qarahe***: **Holotype**, pinned worker, **KSA**: Qaraah village, 18.05°N, 42.75°E, 2,000 m, 16.iv.1976 (Büttiker, W.) (WMLC: CASENT0906344). Paratype, one pinned worker with same data as holotype (WMLC) [examined].

**Non-type material examined**

**CAMEROON**: Nkoemvon, 1980, (Jackson D), (12 w); Nkoemvon, 18.v.1980, (Jackson D), (6 w); Nkoemvon, 04.iii.1980, (Jackson D), (9 w); Mondoni, 14.iii.1990, (Dejean A), (6 w); Yaounde, 26.iii.1989, (Dejean A), (3 w); Mbalmayo, xi.1993, (Stork N), (3 w); **GHANA**: Old Tafo nr. Tafo, 31.i.1992, (Belshaw B), (2 w, 1 q, leaf litter, primary forest); Maabari nr. Tepa, 18.xii.1991, (Belshaw B), (3 w, leaf litter, secondary forest); Asiakwa nr. Kibi, 01.v.1992, (Belshaw B), (3 w, leaf litter Cocoa); Kibi, 24.iii.1970, (Leston D), (17 w, litter samples); Manpong, 03.ii.1970, (Room R), (5 w); Boku, 14.viii.1968, (Collingwood CA), (1 w, on cocoa); Pankese, 30.ix.1968, (Collingwood CA), (1 w, on cocoa); Mampong, 26.i.1970, (Room P), (1 w, 1 q); Legon, A. D., 08.vii.1970, (Leston D), (7 w, 2 q); Tafo, 25.ix.1970, (Bolton B), (6 w, twig on forest floor); Tafo, Cocoa Research Institution, 23.xii.1991, (Belshaw R), (3 w, leaf litter, secondary forest); Tafo, 26.ii.1970, (Bolton B), (2 w, 1 q, log mould); Tafo, 26.ii.1970, (Bolton B), (3 w, log mould samples); Legon, 24.vii.1970, (Leston D), (3 w); Legon, A. D., 08.x.1970, (Leston D), (5 w); Legon, A. D., 14.x.1970, (Leston D), (6 w, 2q); Legon, A. D., 24.vii.1970, (Leston D), (3 w); Tumu, 25.xii.1969, (Room R), (2 w); **KENYA**: Tana R., Kora, 11.vii.1983, (Collins NM, Ritchie M), (6 w, pitfall); **MADAGASCAR**: 48 km ENE, Morondava, 20.066667°S, 44.65°E, 07.i.1991, 30 m, (Olson DM), (2 w, tropical dry forest, CN6T); **NIGERIA:** Black pod project, 05.ii.1975, (Taylor B) (3 w, 1 q); Gambari, 16.vi.1969, (Bolton B), (9 w, on cocoa); Gambari, 20.v.1969, (Bolton B), (7 w, rotten log); Ibadan, 11.x.1987, (Noyes J), (21 w), previous material in BMNH; **OMAN**: Jebel Akhdar, Alain, 23.07279°N, 57.66179°E, 1,949 m, 4.iv.2016, (Sharaf MR) (17 w, KSMA); Jebel Akhdar, Alain, 23.07237°N, 57.66187°E, 1,889 m, 6.iv.2016, (Sharaf MR) (4 w, KSMA); Muscat, 23.62405°N, 58.48891°E, 9 m, 9.iv.2016, (Sharaf MR) (6 w, KSMA); Qurayat, 23.20460°N, 58.96920°E, 39 m, 8.iv.2016, (Sharaf MR) (9 w, KSMA); Date palm, no specific locality, 8.iv.2016, (A Polaszek) (1 w, KSMA); Nakhl, 23.49327°N, 57.83421°E, 190 m, 5.iv.2016, (Sharaf MR) (7 w, KSMA); Muscat, 23.61760°N, 58.49364°E, 81 m, 7.iv.2016, (Sharaf MR) (1 w, KSMA); W. Fanga, 23.46194°N, 58.10326°E, 160 m, 20.i.2017, (Sharaf MR) (3 w, KSMA); Eastern Hajar, Mts, S. side, Samail gap, Lizurgh village, 23.355556°N, 58.105556°E, 280 m, 6.iv.2016, (A Polaszek) (3 w, KSMA); Muscat, KOM, Alraha village, 23.56665°N, 58.17630°E, 74 m, (Sharaf MR) (1 w, KSMA); Masfat Elebryein, 23.14178°N, 57.31330°E, 933 m, 21.i.2017, (Sharaf MR) (3 w, KSMA); Alkhoud village, 23.57154°N, 58.12166°E, 63 m, (Sharaf MR) (1 w, KSMA); W. Fanga, 23.45336°N, 58.11807°E, 166 m, 20.i.2017, (Sharaf MR) (4 w, KSMA); Eastern Hajar, Mnt., Rd to Sur, 23.1675°N, 58.101944°E, 280 m, (A Polaszek) (2 w, KSMA); Western Hajar Mnt., Nakham, 23.388056°N, 57.832222°E, 310 m, 2.iv.2016, (A Polaszek) (12 w, KSMA); Hajar Mnt., Wadi Alkhoud, 23.566667°N, 58.116667°E, 80 m, 13.i.2017, (A Polaszek) (1 w, KSMA); Batinah coast, Hibra village, 23.493733°N, 57.8337°E, 190 m, 5.iv.2016, (A Polaszek) (32 w, KSMA); **KSA: Al Baha Province**: Elqamh park, Baljurshi, 19.913056°N, 41.905°E, 1,931 m, v.2010, (49 w); Elqamh park, Baljurshi, 19.805722°N, 41.711889°E, 1,950 m, ix.2011, (39 w); Amadan, Almandaq, 20.245278°N, 41.468333°E, 1,881 m, v.2010, (21 w); W. Turabah, Almandaq, 20.211028°N, 41.288222°E, 1,793 m, v.2011 (13 w); W. Turabah, Almandaq, 20.241917°N, 41.262833°E, 1,751 m, ix.2011, (6 w); W. Turabah, Almandaq, 20.211028°N, 41.288222°E, 1,793 m, v.2011, (30 w); W. Gonouna, AlUrdiya gov., 19.429361°N, 41.605028°E, 353 m, v.2011, (17 w); Al majardah, W. Khat, 19.08913°N, 41.97126°E, 513 m, xi.2012, (1 w); Al Mukhwah, Dhi Ayn, 19.929417°N, 41.441722°E, 741 m, v.2011, (4 w); Al Mukhwah, Dhi Ayn, 19.93143°N, 41.44185°E, 728 m, iv.2016, (10 w, 2 q); Al Mukhwah, Dhi Ayn, 19.92967°N, 41.44291°E, 706 m, iv.2016, (2 w); Al Mukhwah, Dhi Ayn, 19.929417°N, 41.441722°E, 741 m, v.2010, (25 w); Al Mukhwah, Dhi Ayn, 19.92967°N, 41.44291°E, 706 m, ix.2011, (3 w); Al Mukhwah, Dhi Ayn, 19.92967°N, 41.44291°E, 744 m, ix.2011, (11 w); Al Mukhwah, Dhi Ayn, 19.92967°N, 41.44291°E, 741 m, v.2011, (3 w); Shada Al A’la, 19.845167°N, 41.30445°E, 741 m, i.2015, (1 w); Shada Al A’la, 19.842917°N, 41.311517°E, 1,666 m, xi.2015, (1 w); W. Elzaraeb, 20.073417°N, 41.38675°E, 2,086 m, v.2011, (1 w), previous material is collected by Sharaf, MR and deposited in KSMA; Shada Al A’la, 19.842917°N, 41.311517°E, 1,666 m, xii.2014, (2 w); Shada Al A’la, 19.842917°N, 41.311517°E, 1,666 m, vii.2015, (2 w); Shada Al A’la, 19.845167°N, 41.30445°E, 1,474 m, iii.2015, (1 w); Shada Al A’la, 19.840183°N, 41.311433°E, 1,611 m, vii.2015, (1 w); Shada Al A’la, 19.8511°N, 41.300617°E, 1,325 m, iii.2015, (1 w); Shada Al A’la, 19.842917°N, 41.311517°E, 1,666 m, xi.2015, (1 w), previous material is collected using PT by Al Dhafer et al. **Asir Province**: Dalaghan park, 18.066066°N, 42.710981°E, 2,223 m, iv.2008, (2 w); Abha-Khamis Mushayt Road, 18.239186°N, 42.588481°E, 2,129 m, ix.2004, (5 w); W. Bagara, 18.79214°N, 42.01912°E, 428 m, vi.2014, (3 w); Abha-Khamis Mushayt Road, 18.23875°N, 42.570694°E, 2,147 m, iv.2011, (21 w); Abha-Khamis Mushayt Road, 18.23875°N, 42.570694°E, 2,147 m, x.2014, (12 w); Raydah: 18.204267°N, 42.4124°E, 2820 m, ii.2014, (12 w); 18.204267°N, 42.4124°E, 2,820 m, iv.2014, (4 w); 18.22217°N, 42.40241°E, 2,744 m, vi.2014, PT (4 w), previous materials are collected by Sharaf, MR; Raydah: 18.204267°N, 42.4124°E, 2,820 m, vi.2014, (1 w); 18.204267°N, 42.4124°E, 2,820 m, viii.2014, (4 w); 18.204267°N, 42.4124°E, 2,820 m, iii.2015, (2 w); 18.20525°N, 42.410117°E, 2,761 m, ii.2014, (1 w); 18.20525°N, 42.410117°E, 2,761 m, iv.2014, (3 w); 18.20525°N, 42.410117°E, 2,761 m, vi.2014, (3 w); 18.20525°N, 42.410117°E, 2,761 m, viii.2014, (2 w); 18.20525°N, 42.410117°E, 2,761 m, i.2015, (1 w); 18.20525°N, 42.410117°E, 2,761 m, i.2015, PT (2 w); 18.20525°N, 42.410117°E, 2,761 m, vii.2015, (3 w); 18.20525°N, 42.410117°E, 2,761 m, xi.2015, (2 w); 18.198067°N, 42.40725°E, 2,387 m, ii.2014, (4 w); 18.198067°N, 42.40725°E, 2,387 m, iv.2014, (1 w); 18.198067°N, 42.40725°E, 2,387 m, 26.viii.2014, (4 w); 18.198067°N, 42.40725°E, 2,387 m, x.2014, (3 w); 18.198067°N, 42.40725°E, 2,387 m, xii.2014, (1 w); 18.198067°N, 42.40725°E, 2,387 m, iii.2015, (4 w); 18.198067°N, 42.40725°E, 2387 m, iv.2015, (4 w); 18.198067°N, 42.40725°E, 2,387 m, vii.2015, (3 w); 18.201583°N, 42.408933°E, 2,578 m, ii.2014, (5 w); 18.201583°N, 42.408933°E, 2,578 m, iv.2014, (3 w); 18.201583°N, 42.408933°E, 2,578 m, vi.2014, (7 w); 18.201583°N, 42.408933°E, 2,578 m, viii.2014, (10 w); 18.201583°N, 42.408933°E, 2,578 m, x.2014, (2 w); 18.201583°N, 42.408933°E, 2,578 m, iii.2015, (1 w); 18.201583°N, 42.408933°E, 2,578 m, v.2015, (5 w); 18.201583°N, 42.408933°E, 2,578 m, vii.2015, (5 w); 18.201583°N, 42.408933°E, 2,578 m, xi.2015, (2 w); 18.1961°N, 42.40525°E, 2,285 m, ii.2014, (2 w); 18.1961°N, 42.40525°E, 2,285 m, viii.2014, (3 w); 18.1961°N, 42.40525°E, 2,285 m, x.2014, (2 w); 18.1961°N, 42.40525°E, 2,285 m, xi.2015, (2 w); 18.1961°N, 42.40525°E, 2,285 m, iii.2015, (5 w); 18.1961°N, 42.40525°E, 2,285 m, i.2015, (1 w); 18.1961°N, 42.40525°E, 2,285 m, v.2015, (3 w); 18.194917°N, 42.396967°E, 1,897 m, x.2014, (5 w); 18.194917°N, 42.396967°E, 1,897 m, iii.2015, (1 w); 18.194917°N, 42.396967°E, 1897 m, vii.2015, (8 w); 18.194917°N, 42.396967°E, 1851 m, xii.2014, (1 w); 18.195817°N, 42.389083°E, 1614 m, ii.2014, (1 w); 18.195817°N, 42.389083°E, 1,614 m, viii.2014, (5 w); 18.195817°N, 42.389083°E, 1,614 m, xii.2014, (5 w); 18.195817°N, 42.389083°E, 1,614 m, i.2015, (2 w); 18.195817°N, 42.389083°E, 1,614 m, iii.2015, (3 w); 18.195817°N, 42.389083°E, 1,614 m, vii.2015, (1 w); 18.195817°N, 42.389083°E, 1,614 m, ix.2015, (3 w); 18.195817°N, 42.389083°E, 1,614 m, xi.2015, (3 w); 18.193633°N, 42.390333°E, 1,772 m, iv.2014, (1 w); 18.193633°N, 42.390333°E, 1,772 m, viii.2014, (1 w); 18.193633°N, 42.390333°E, 1,772 m, x.2014, (2 w); 18.193633°N, 42.390333°E, 1,772 m, xii.2014, (1 w); 18.193633°N, 42.390333°E, 1,772 m, i.2015, (1 w); 18.193633°N, 42.390333°E, 1,772 m, iii.2015, (1 w); 18.193633°N, 42.390333°E, 1,772 m, v.2015, (1 w); 18.193633°N, 42.390333°E, 1,772 m, vi.2015, (3 w); 18.193633°N, 42.390333°E, 1,772 m, vii.2015, (1 w); 18.193633°N, 42.390333°E, 1,772 m, ix.2015, (2 w), previous material is collected using PT by Al Dhafer et al. and deposited in KSMA; **Riyadh Province:** Riyadh, Takhassosy street, 24.693472°N, 46.671136°E, 615 m, x.2015, (1 w); Riyadh, Alhayer, 24.280194°N, 46.765861°E, iii.2009, (6 w); Dirab, KSU research farm, 24.4085°N, 46.661639°E, 588 m, xii.2009, (1 w); Oyaina, 24.90665°N, 46.389917°E, 749 m, iv.2010, (3 w); Qarina, 25.132275°N, 46.163883°E, 761 m, (3 w); Riyadh, Al Emam University campus, 24.809158°N, 46.701892°E,650 m, x.2010, (1 w); Riyadh, KSU guest building, Dereyia, 24.716667°N, 46.616667°E, 612 m, vii.2009, (1 w), previous material is collected by Sharaf, MR and deposited in KSMA; Riyadh, Janaderiyah, 24.98121°N, 46.77711°E, 630 m, ix.2014, (1 w); Riyadh, Hawtet Sudair, 25.60490°N, 45.60050°E, 751 m, i.2015, (1 w); Hawtet Bani Tamim, 23.48019°N, 46.84350°E, 597 m, i.2014, (3 w); Hawtet Bani Tamim, 23.50737°N, 46.90059°E, 593 m, xii.2014, (10 w); Hawtet Bani Tamim, 23.45943°N, 46.81895°E, 582 m, xii.2014, (4 w); Riyadh, Ammariya, 24.81839°N, 46.44698°E, 696 m, x.2013, (11 w); Mezahmiya, 24.45570°N, 46.14881°E, 719 m, x.2013, (42 w); Mezahmiya, 24.47197°N, 46.23878°E, 633 m, i.2014, (2 w); Dirab, KSU research farm, 24.4085°N, 46.661639°E, 588 m, xii.2013, (4 w); Layla (Alaflaj), 22.21325°N, 46.68818°E, 543 m, i.2014, (4 w); Elsolayel, 20.45473°N, 45.57120°E, 616 m, i.2014, (2 w); Riyadh, W. Hanifa, 24.67089°N, 46.58061°E, 654 m, ii.2014, (3 w); Riyadh, W. Hanifa, 24.77091°N, 46.53147°E, 695 m, ix.2014, (10 w); Riyadh, W. Hanifa, 24.74747°N, 46.56474°E, 679 m, ix.2014, (4 w); Riyadh, W. Hanifa, 24.73507°N, 46.57518°E, 674 m, ix.2014, (2 w); Huraymila, 25.12636°N, 46.15782°E, 951 m, ii.2014, (2 w); Dhurma, 24.59961°N, 46.15547°E, 657 m, iv.2014, (2 w); Thadiq, 25.30974°N, 45.86457°E, 736 m, iv.2014, (2 w); Thadiq, 25.29360°N, 45.87102°E, 735 m, iv.2014, (2 w); Quwaiiyah, 24.04718°N, 45.24430°E, 854 m, v.2014, (3 w); Quwaiiyah, 24.04347°N, 45.24007°E, 857 m, v.2014, (3 w); Shaqra, 25.26655°N, 45.26779°E, 728 m, v.2014, (2 w); Shaqra, 25.32638°N, 45.23341°E, 710 m, v.2014, (2 w); Shaqra, 25.23018°N, 45.31915°E, 703 m, i.2015, (3 w); Al Majma’a, 25.93294°N, 45.29779°E, 736 m, vi.2014, (3 w); Al Majma’a, 25.92384°N, 45.38035°E, 743 m, ii.2014, (5 w); Al Ghat, 26.02495°N, 44.93650°E, 670 m, vi.2014, (3 w); Al Ghat, 26.06582°N, 44.91929°E, 653 m, x.2015, (2 w); Riyadh-Al Kharj Road, 24.29615°N, 47.15553°E, 453 m, ix.2014, (4 w); W. Al Dawasir, 20.49055°N, 44.79462°E, 721 m, ii.2015, (2 w), previous material is collected by Salman, S. and deposited in KSMA; **Jazan Province:** Abu Arish, 17.01347°N, 42.80160°E, 90 m, iv.2012, (6 w); W. Shahdan, 17.45222°N, 42.71516°E, 200 m, xi.2012, (5 w); W. Aljora near Abadan, 17.29263°N, 43.07010°E, 465 m, xi.2012, (7 w). Eastern Province: Al Qatif, El Naft, 26.510278°N, 49.968889°E, 30 m, iii.2012, (2 w) All previous material is collected by Sharaf MR and deposited in KSMA; Wadi Shugub, 1,390 m, iv.1983, (1 w); Al Tawlah, 1.iv.1983, (1 w); Annamas, 8.iv.1983, (1 w); Hufuf, 14.iv.1983, (1 w), previous material is collected by CA Collingwood and deposited in WMLC; **TANZANIA**: Kizimgani, 16.iii.1988, (MJ Ways), (3 w); Sotale, x.1989, (Lohr), (2 w); Kanga, x.1989, (Lohr), (1 w).**UNITED ARAB EMIRATES**: Khor al-khwair, 25.95°N, 56.05°E, 08.iii-07.v.2007, (M Hauser et al.), UAE13173, CASENT0264114 (1 w, KSMA); Sharjah, 25.35°N, 55.4°E, 28.ii-12.iv.2011, (M Hauser et al.), UAE13028, CASENT0264785 (1 w, KSMA); W. Bih dam, 25.8°N, 56.066667°E, 16-31.xii.2009, (M Hauser et al.), UAE13123, CASENT0264962 (1 w, KSMA); Dubai, palm Deira trees by metro, 25.276°N, 55.300°E, 16.v.2012, (Wetterer J), (2 w, #99, #107, KSMA); no locality, iii.2005, (Collingwood ), UAE173, (1 w, WMLC); **ZIMBABWE**: Bulawayo, 04.xii.1916, (G Arnold) (18 Syntype w); Sawmills, 22.xi.1918, (G Arnold), (3 w, 2 m); Victoria Falls, 09.xii.1914, (G Arnold), (7 w).

**Description**

**Head.** In full-face view distinctly longer than broad with nearly parallel sides and feebly concave or straight posterior margin; clypeal carinae feebly developed, broadly separated and clearly divergent anteriorly; anterior clypeal margin feebly concave, without anterior sharp angles; antennae 11-segmented; scapes, when laid back straight from their insertions, failing to reach posterior margin of head; eye size variable (EL 0.19–0.22 × HW), in profile consisting of an outer ring of ommatidia encloses one or two longitudinal rows of 2–4 ommatidia; in few specimens one or two ommatidia within the ring present; eyes distinctly situated in front of midlength of head sides in full-face view. **Mesosoma.** Promesonotum feebly convex in profile; metanotal groove distinctly impressed, with short cross-ribs; propodeal dorsum and declivity meeting in a rounded convexity. **Petiole.** Petiolar peduncle short, with a small anteroventral process in profile; petiolar node low-subconical in profile. **Postpetiole.** Postpetiole smaller than petiole, lower and broadly convex dorsally in profile. **Sculpture.** Entire body smooth and shining, except for metanotal cross-ribs on sides of metanotal groove. **Pilosity.** All body surfaces with standing hairs, pronotum with a single pair on anterior margin between humeral pair; promesonotum usually with four pairs of hairs but in many specimens a fifth pair present; propodeum without hairs or with one or two pairs of hairs. **Color.** Variable, from uniform yellow to uniform dark brown, frequently with a pair of brown patches or a darker band apically on first gastral tergite.

**Note**

Aldawood & Sharaf (2011) described *M. dryhimi* from Al Baha Province (KSA) based on the worker caste. Comparing the type material of *M. dryhimi* and *M. montanum* with *M. exiguum* revealed that they have similar body size and color, in addition to the possession of the following characters: eyes of moderate size, with an outer ring of ommatidia encloses one or two longitudinal rows of 2–4 ommatidia; metanotal groove impressed, with distinct cross-ribs; all body surfaces with standing hairs, pronotum with a single pair on anterior margin between humeral pair; posterior half of first gastral tergite brown; body smooth and shining. Herein, we treat *M. dryhimi* and *M. montanum* as junior synonyms of *M. exiguum*.

**Biological and ecological notes**

This species is by far the most common Arabian species of the *M. monomorium*-group, and it appears to be very flexible in its ecological requirements since it occurs in numerous habitats throughout the Arabian Peninsula. It was found living in humid soil, leaf litter, under rocks, and under bark. Once it was even collected from inside galleries of a *Camponotus* sp. colony. Furthermore, *M. exiguum* was found in a variety of agricultural landscapes and human settlements, very often in close proximity to trees or other vegetation.

**Geographic range**

Even though this species was originally described from Ethiopia (Forel, 1894), *M. exiguum* is extremely widespread (Table 1) since it occurs in much of the Afrotropical Region (Bolton, 1987; Sharaf et al., 2017), the Malagasy Region (Heterick, 2006), and the Mediterranean Basin (Gòmez & Espadaler, 2006; Sharaf, 2006). As mentioned above, this species is also broadly distributed throughout the Arabian Peninsula, and is considered as the commonest species of the *M. monomorium*-group in the region. The first record from the Arabian Peninsula was from the KSA (Aldawood & Sharaf, 2009) while later records from the UAE, Oman, and Yemen (Collingwood et al., 2011) were published under the name *M. baushare* (Collingwood & Agosti, 1996) that was recently synonymized under *M. exiguum* (Sharaf et al., 2017).

**Table:** 

**Figure 8:**
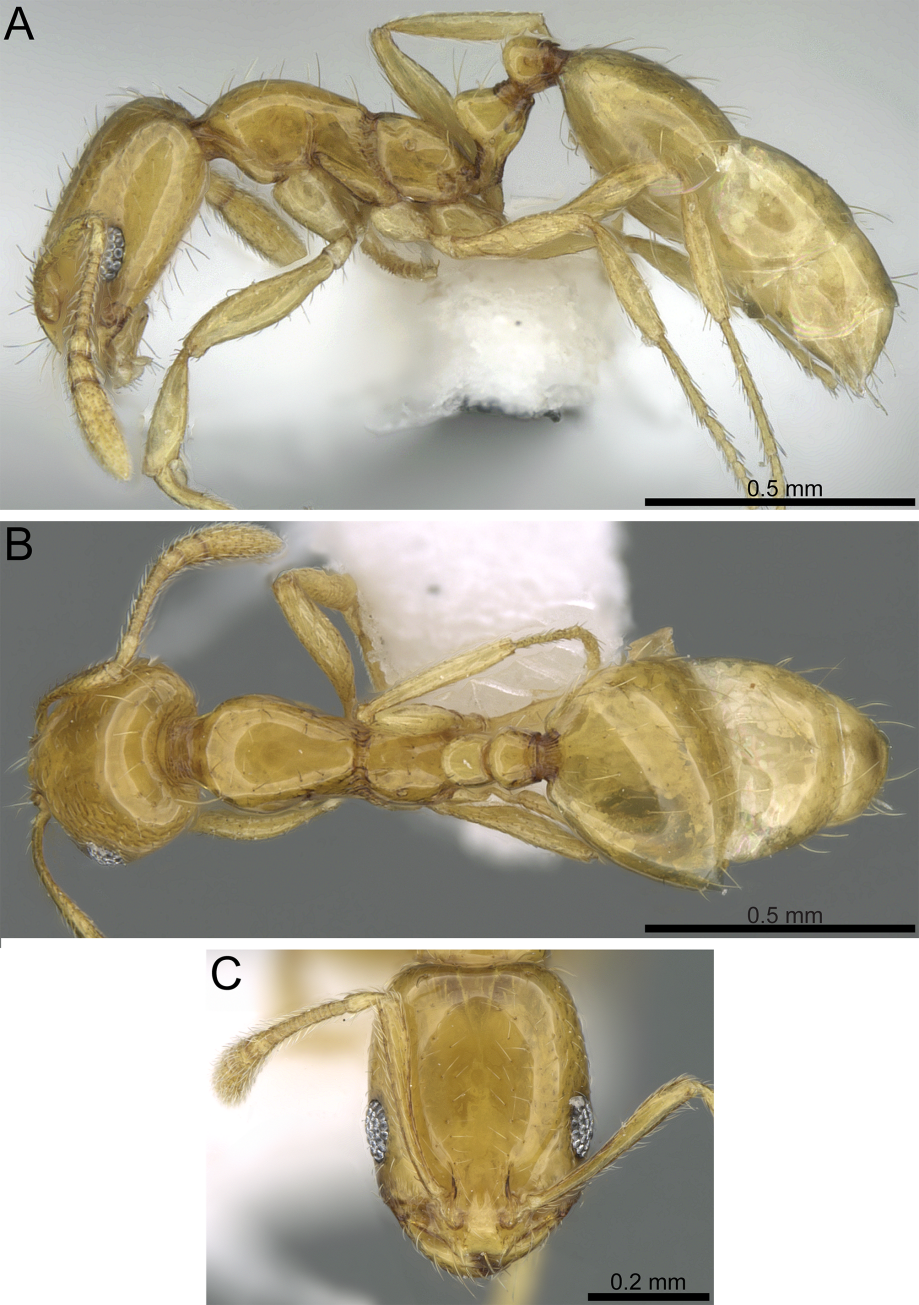
*Monomorium holothir* (CASENT0906392, from https://www.antweb.org/specimenImages.do?name=casent0906392&countryName=Seychelles, photographer: Estella Ortega). (A) Body in profile. (B) Body in dorsal view. (C) Head in full-face view.

***Monomorium holothir*** **Bolton, 1987**, **Fig. 8**
*Monomorium holothir* Bolton, 1987: 393 (w.)

**Type material examined**

**Holotype**, pinned worker, **KENYA**: Lake Baringo, 1.xii.1983 (L. Darlington) (BMNH: CASENT0902243). **Paratype**, pinned worker with same data as holotype (BMNH).

**Non-type material examined**

**KSA**: Jazan, 16.97627°N, 42.61743°E, 38 m, 12.iv.2012, (Sharaf MR) (4 w, KSMA) (CASENT0906392); Jazan, Abu Arish, 17.01347°N, 42.80160°E, 90 m, 10.iv.2012, (Sharaf MR) (1 w, KSMA); Jazan, Zabia, 17.10745°N, 42.65026°E, 43 m, 9.iv.2012, (Sharaf MR) (4 w, KSMA).

**Description**

**Head.** In full-face view distinctly longer than broad with feebly convex sides behind eyes and nearly straight or feebly concave posterior margin; clypeal carinae sharply developed; anterior clypeal margin feebly concave; antennae 12-segmented; scapes failing to reach posterior margin of head; eyes relatively large (EL 0.30 × HW), with 7–9 ommatidia in longest row; in profile eye length clearly greater than distance between anteriormost point of eyes and nearest point of mandibular insertion. **Mesosoma.** Promesonotum feebly convex in profile; metanotal groove distinctly impressed, with distinct cross-ribs; propodeal dorsum and declivity meeting in a rounded convexity; propodeal spiracle small and pinhole-like. **Petiole.** Petiolar node high, subconical and with narrowly rounded dorsum in profile. **Sculpture.** Entire body smooth and shining, except for metanotal cross-ribs on sides of metanotal groove. **Pilosity.** All body surfaces with abundant long-standing hairs; promesonotum with more than six pairs of hairs; propodeum with four pairs of hairs. **Color.** Variable, from yellow to brown-yellow.

**Biological and ecological notes**

This species was collected from mangos, *Mangifera* sp. (Anacardiaceae) imported from Kenya and this provides the context for the present record. It was also found in leaf litter next to *Calotropis procera* (Aiton) W.T.Aiton (Asclepiadaceae). Some workers were found nesting in a thin layer of clay soil above sandy soil, while several workers were collected from leaf litter under a *Conocarpus* L. tree (Combretaceae).

**Geographic range**

*Monomorium holothir* is a comparatively rare species originally described from Kenya and prior to this study only known from the type locality (Bolton, 1987; Hita Garcia, Wiesel & Fischer, 2013). Our collections represent a new species record for KSA, and it is highly likely an introduction to the country.

**Table:** 

**Figure 9:**
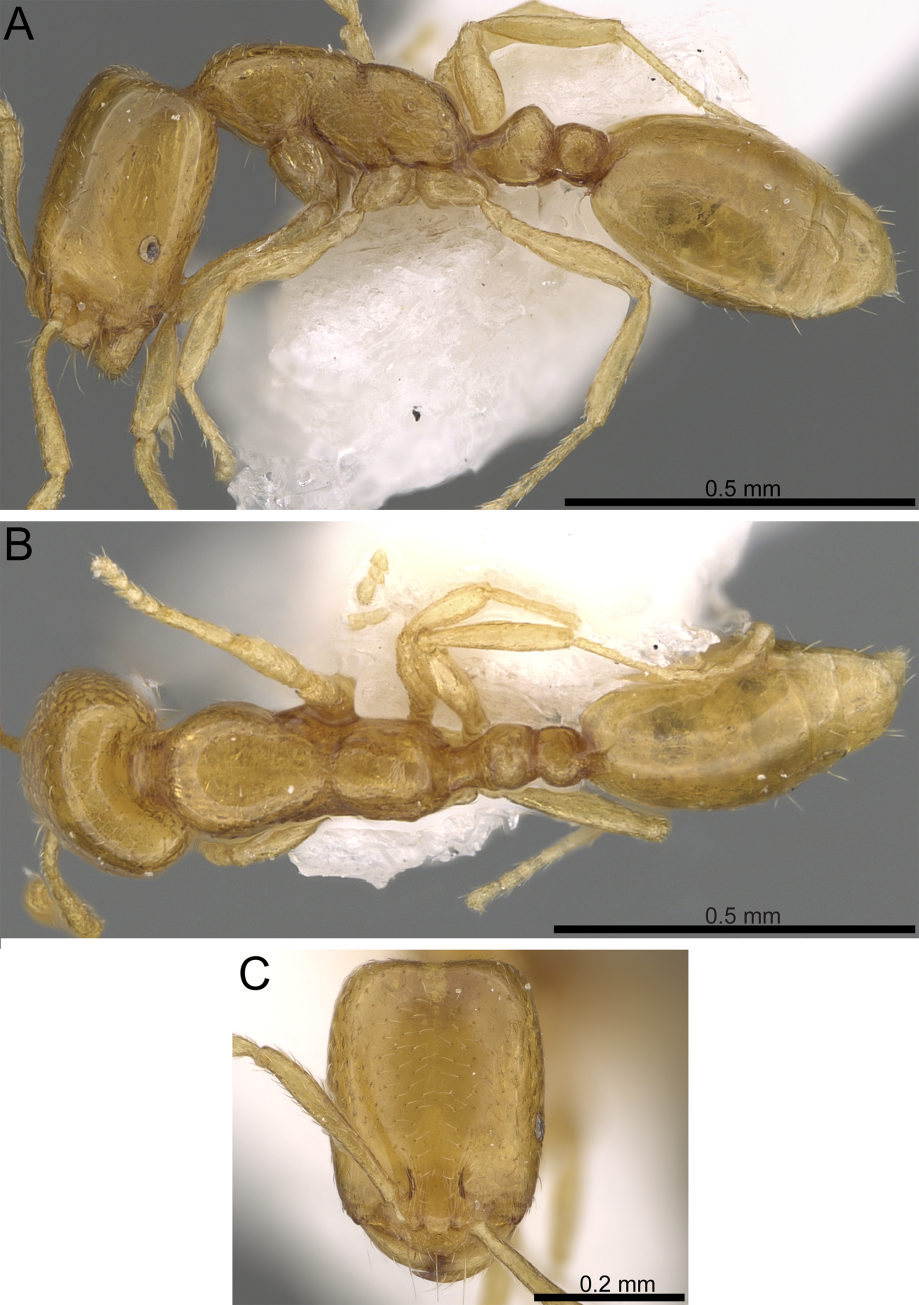
*Monomorium mohammedi* **sp. n.** (CASENT0922351, from https://www.antweb.org/specimen.do?name=casent0922351, photographer: Michele Esposito). (A) Body in profile. (B) Body in dorsal view. (C) Head in full-face view.

***Monomorium mohammedi*** **Sharaf & Hita Garcia sp. n., Fig. 9**

**Type material**

**Holotype**, pinned worker, **KSA**: Almajardah, Wadi Khat, 19.08913°N, 41.97126°E, 513 m, 10.xi.2012, (Sharaf MR) (KSMA: CASENT0823774). **Paratypes**, six pinned workers: **KSA**: 2 w, with same data as the holotype (KSMA); 3 w, Jazan, Wadi Shahdan, 17.45222°N, 42.71516°E, 200 m, 13.xi.2012, (Sharaf MR) (1 in CASC: CASENT0922351, 2 in KSMA); 1 w, Jazan, Abu Arish, 17.01347°N, 42.80160°E, 90 m, 10.iv.2012, (Sharaf MR) (WMLC).

**Measurements**

**Holotype**: TL 1.37; HL 0.41; HW 0.31; SL 0.25; EL 0.04; EM 0.09; ML 0.42; PW 0.19; PTL 0.15; PTW 0.08; PTH 0.11; PPL 0.07; PPW 0.08; PPH 0.08; CI 76; EI 13; SI 81.

**Paratypes** (*n* = 7): TL 1.32–1.53; HL 0.38–0.44; HW 0.29–0.32; SL 0.26–0.29; EL 0.04–0.05; EM 0.09–0.11; ML 0.36–0.44; PW 0.18–0.21; PTL 0.08–0.11; PTW 0.07–0.08; PTH 0.09–0.12; PPL 0.05–0.07; PPW 0.08; PPH 0.08; CI 71–82; EI 13–17; SI 88–97.

**Diagnosis.** *Monomorium mohammedi* can be readily diagnosed by the combination of the following characters: eyes distinctly small, with 5–6 ommatidia; mesosoma, petiole, and postpetiole without standing hairs; mesopleuron, metapleuron, petiole and postpetiole finely shagreened.

**Description**

**Worker. Head**. In full-face view distinctly longer than broad with shallowly convex or nearly parallel sides and clearly concave posterior margin in full-face view; median clypeal portion without carina or anterolateral angles, anterior clypeal margin feebly concave; antenna 11-segmented; scapes short, when laid straight back, just surpassing midlength of head (SI 88–97); mandibles armed with three teeth, decreasing in size from apex to base; eyes oval, tiny, (EL 0.13–0.15 × HW) with 5 ommatidia, set in front of midlength of head; frontal lobes farther apart in full-face view; underside of head with six scattered short hairs. **Mesosoma.** In profile with a flat promesonotal dorsum, which slopes posteriorly to a well-defined metanotal groove; propodeal spiracles small and pinhole-like; propodeal dorsum evenly sloping posteriorly to short declivity. **Petiole.** Node massive, narrowly rounded above, and little higher than postpetiolar node in profile; anterior peduncle short; ventral petiolar surface below node broadly convex extending anteriorly to form a blunt broad dent. **Postpetiole**. Node small with convex dorsal margin; postpetiole as high as broad. **Sculpture.** Cephalic surface smooth and shining; mandibles smooth and shining, with faint striations on the outer margin; mesosoma dorsum and propleuron smooth and shining; meso-and metapleuron finely shagreened; metanotal cross ribs distinct; petiole and postpetiole with traces of superficially shagreened sculpture, but never smooth; gaster smooth and shining. **Pilosity**. Underside of head without hairs; cephalic surface with scattered minute hair-pits; anterior clypeal margin and mandibles with longer hairs; antennae with abundant appressed hairs; mesosoma without hairs, only rare appressed pubescence; petiole and postpetiole without hairs, only few appressed pubescence dorsally; gaster with scattered appressed pubescence, few longer hairs on the last gastral tergites. **Color**. Overall uniform clear yellow, mandibular teeth light brown.

**Differential diagnosis**

This new species is closest to *M. guillarmodi* Arnold, 1946 from Lesotho in terms of the small body size, tiny eyes, 11-segmented antennae, lack of hairs on mesosoma, and the smooth body. However, *M. mohammedi* is readily separated from *M. guillarmodi* by the following characters: the posterior margin of head without hairs and concave in full-face view, petiole and postpetiole without hairs, median clypeal portion without anterolateral angles or carina, scape relatively longer (SI 81–97), whereas *M. guillarmodi* has a transverse posterior head margin with 1–2 pairs of hairs, petiole and postpetiole each with a single pair of hairs, median clypeal portion prominent with well-defined anterolateral angles and distinct carina, and scape shorter (SI less than 80).

Among the Arabian species of the *M. monomorium*-group four species have 11-segmented antennae: *M. mohammedi*, *M. clavicorne*, *M. aeyade*, and *M. exiguum*. *Monomorium mohammedi* is easily separated from *clavicorne* by its smaller eyes, smaller terminal funicular segment, and lack of mesosomal pilosity, whereas *clavicorne* has larger eyes, greatly swollen terminal funicular segments, and abundant hairs on the mesosoma.

When comparing *M. mohammedi* with *M. aeyade*, both lack hairs on the mesosoma but *M. mohammedi* can be immediately separated by its smaller eyes with only 5 ommatidia that are situated distinctly farther apart from the mandibular insertions (EM 0.09–0.11), the finely shagreened meso- and metapleuron, the hairless petiole and postpetiole, whereas *M. aeyade* has larger eyes (EL 0.24 × HW), with a ring of ommatidia encircling a single row of 2 ommatidia that are situated closer to the mandibular insertions (EM 0.05); meso-and metapleuron smooth, and petiole and postpetiole each with one pair of hairs.

**Biological and ecological notes**

The new species was collected from leaf litter under a *Hyphaene* tree (Arecaceae) and another nest series was found in a thin layer of clay soil above sandy soil under a Mango tree.

**Geographic range**

So far, the new species is only known from the type locality.

**Etymology**

The name of the new species is a patronym in honor of Mohammed Sharaf, the eight year old son of the senior author.

**Table:** 

***Monomorium sarawatense* Sharaf & Aldawood, 2013, Fig. 10**
*Monomorium sarawatensis* Sharaf & Aldawood, 2013: 70, figs. 5–15 (w).

**Type material examined**

**Holotype**, pinned worker, **KSA**: Al-Baha Province, Aqabet Al-Baha-Tihama, 20.00000°N, 41.43758°E, 1,300 m, 19.iv.2012 (Sharaf MR) (KSMA). **Paratypes**, 26 pinned workers with same data as holotype (one in BMNH, 25 in KSMA).

**Figure 10:**
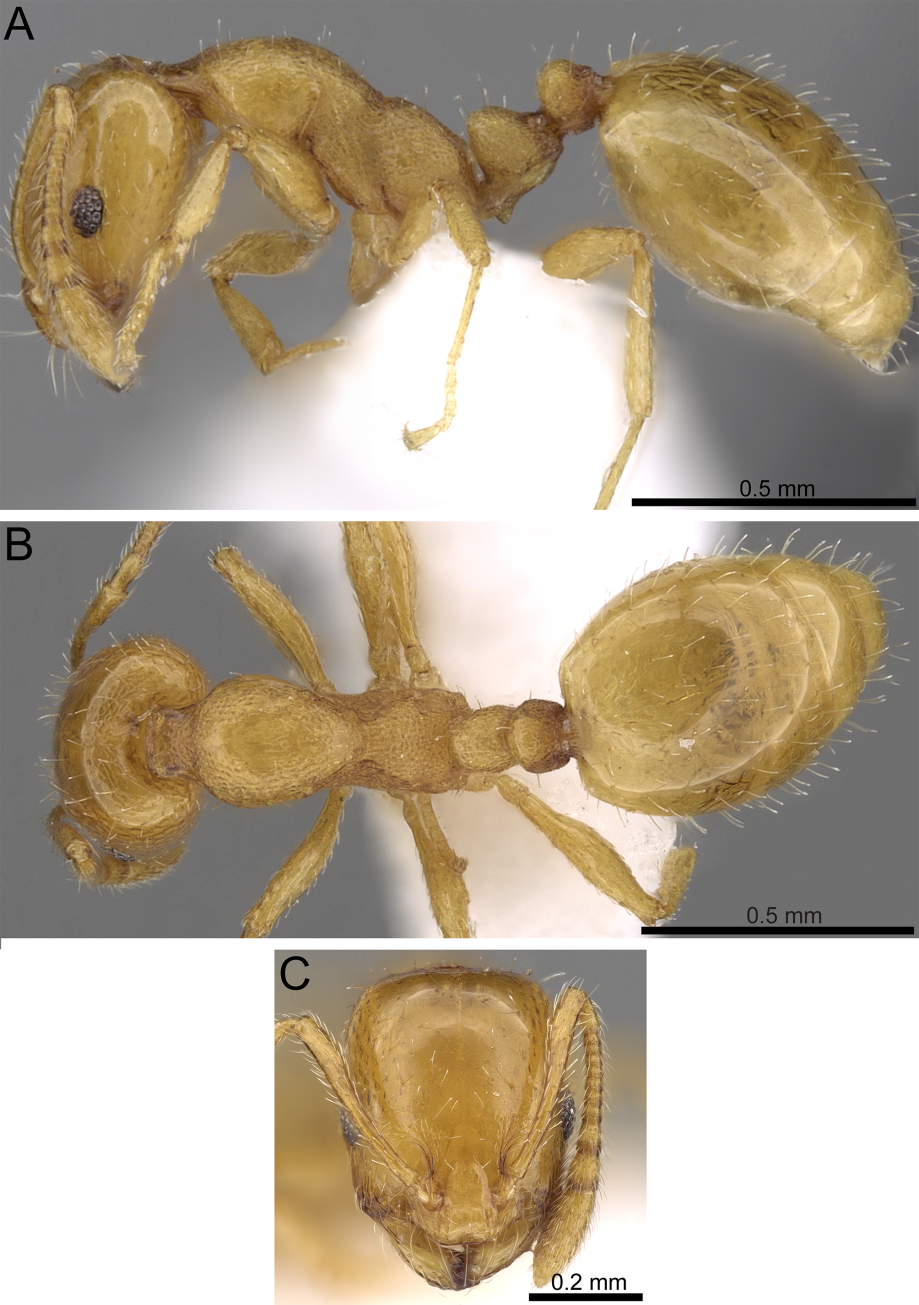
*Monomorium sarawatense* (CASENT0280971, from https://www.antweb.org/specimen.do?name=CASENT0280971, photographer: Estella Ortega). (A) Body in profile. (B) Body in dorsal view. (C) Head in full-face view.

**Non-type material examined**

**KSA**: Al Baha Province, Aqabet Al-Baha-Tihama, 20.00000°N, 41.43758°E, 1,300 m, 19.iv.2012 (Sharaf MR) (21 w, KSMA); Shada Al A’la, 19.8511°N, 41.300617°E, 1325 m, 2.iii.2015, (Al Dhafer et al.) (1 w, KSMA); Asir Province: Raydah, 18.1961°N, 42.40525°E, 2285 m, 26.iii.2014, Malaise trap (Al Dhafer et al.) (1 w, KSMA).

**Description**

**Head**. Distinctly longer than broad, with nearly straight posterior margin and shallowly convex sides; anterior clypeal margin feebly concave between a pair of obtusely projecting angles; clypeal carinae broadly separated and subparallel; antenna 12-segmented; scapes, when laid back from their insertions, fail to reach posterior margin of head; eyes with five-six ommatidia in longest row (EL 0.17–0.22 × HW); with head in profile the posterior margins of eyes at midlength of sides. **Mesosoma**. In profile with promesonotum straight or feebly convex; metanotal groove deep and broad; propodeal dorsum making weak obtuse angle with propodeal declivity; propodeal spiracle small and pinhole-like. **Petiole.** Petiolar node high and acuminate in profile, petiolar peduncle thick and short. **Postpetiole**. In dorsal view clearly broader than long. **Sculpture.** Cephalic dorsum smooth and shining; genae faintly longitudinally striate; mesosoma densely reticulate-punctate except for pronotal sides, which are nearly smooth and shining; petiole and postpetiole densely reticulate-punctate. **Pilosity**. Body pilosity clubbed; cephalic dorsum with few scattered hair-pits; mesosomal pilosity few and sparse, two pairs of erect setae on pronotum, five or more on mesonotum, three on propodeum, petiole usually with two pairs of erect setae. **Color**. Uniformly yellow.

**Biological and ecological notes**

*Monomorium sarawatense* was found nesting inside woody fruits of *Annona squamosal* L. (Annonaceae).

**Geographic range**

Based on current knowledge, this species is endemic to the southwestern Mountains of the KSA.

## Discussion

The previously documented number of species of the *M. monomorium*-group from the Arabian Peninsula was six: *M. aeyade*, *M. clavicorne*, *M. dryhimi*, *M. exiguum*, *M. montanum*, and *M. sarawatense* (Collingwood, 1985; Collingwood & Agosti, 1996; Aldawood & Sharaf, 2009; Aldawood & Sharaf, 2011; El-Hawagryi et al., 2013; Sharaf et al., 2015; Sharaf et al., 2017). In this study, the number of species remains six due to some taxonomic amendments, additional records, and the description of *M. mohammedi* sp. n.

**Figure 11:**
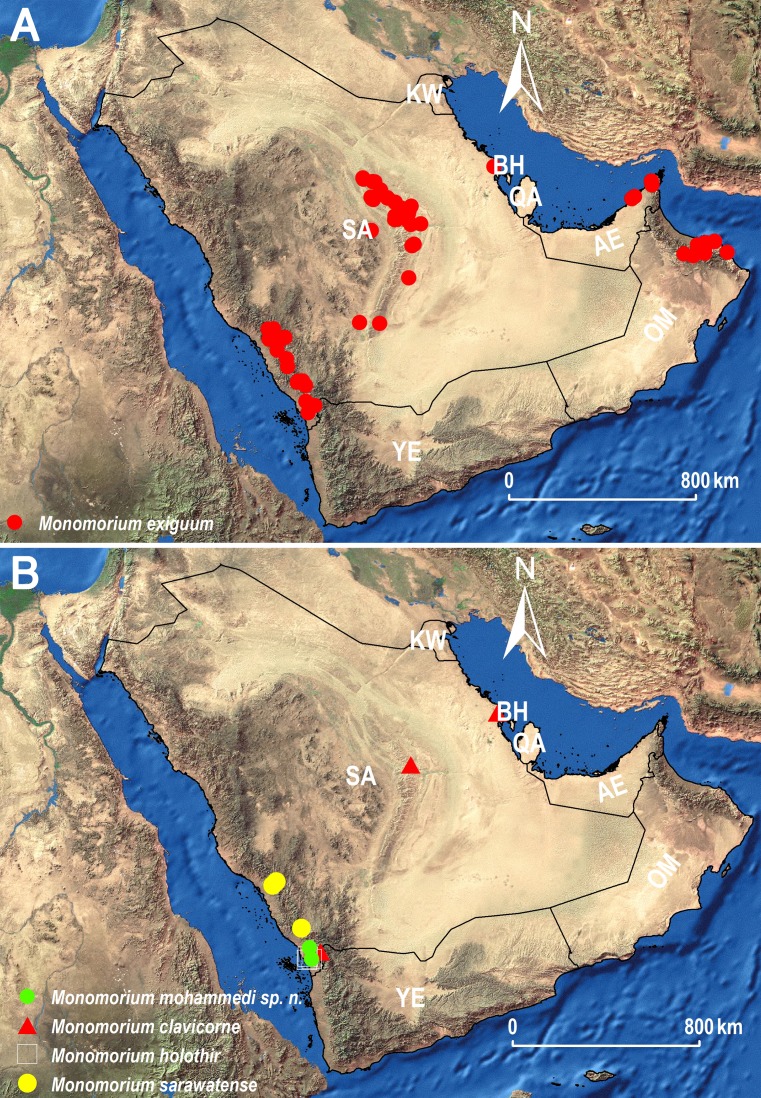
Distribution maps showing the known distribution ranges of the treated species on the Arabian Peninsula, except for *M. aeyade* Collingwood & Agosti, for which no exact locality data exists. (A) Distribution range of *M. exiguum* Forel (red circles). (B) Distribution ranges of *M. clavicorne* André (red triangles), *M. holothir* Bolton (white squares), *M. mohammedi* **sp. n.** (green circles), and *M. sarawatense* Sharaf & Aldawood (yellow circles), AE: United Arab Emirates, BA: Bahrain, KW: Kuwait, OM: Oman, QA: Qatar, SA: Saudi Arabia, YE: Yemen.

Within the *M. monomorium*-group, *M. exiguum* is the most broadly distributed species (Fig. 11A) due to its ability to inhabit a broad range of habitats in the region. The species is widespread in the central and southwestern regions of the KSA, Oman and the UAE but it is highly likely that it has a similar broad geographical distribution in other unexplored areas of the Arabian Peninsula. In addition, the species has a wide distribution range in the Afrotropical region (Table 1). The recent synonymies of some Arabian species under *M. exiguum* (Sharaf et al., 2017), as well as the ones suggested in this study, reveal a remarkable size and color variation, which has led to erroneous species descriptions in the past. During the present work, it was observed that some nest series are uniform yellow, or with brown transverse bands on the first and second gastral tergites, whereas other series have brown heads and yellow-brown gasteral tergites. These variations were already noted by Bolton (1987) for the Afrotropical fauna. These morphological variations should be taken into consideration in future studies that will treat the *M. monomorium*-group in the region, especially those concerning descriptions of new species, in order to avoid possible synonymies. Considering the challenging morphological species delimitations, integrative approaches combining morphological and molecular techniques might provide a more comprehensive taxonomic system (Schlick-Steiner et al., 2010).

The distribution of the three species *M. holothir*, *M. mohammedi*, and *M. sarawatense* (Fig. 11B) appears to be confined to the southwestern region of the KSA. The latter region is known for its Afrotropical affinities (Bolton, 1994; Eig, 1938; Zohary, 1973; Aldawood, Sharaf & Taylor, 2011; Sharaf & Aldawood, 2012; Sharaf, Aldawood & El-Hawagry, 2012a; Sharaf, Aldawood & El-Hawagry, 2012b; El-Hawagryi et al., 2013; Sharaf & Aldawood, 2013), and the newly recorded *Monomorium holothir*, a species previously only known from Kenya, emphasizes this Afrotropical influence.

We hope that this work will help regional researchers with the identification of this rather challenging group of ants and we also expect adding more material including new records and undescribed species with further surveys of poorly collected areas of the region.

### Conclusions

The Arabian ant fauna of the *Monomorium monomorium* species-group is revised, keyed and illustrated based on the worker caste. Six species are treated with description of a new species *M. mohammedi* sp. n. from the southwestern region of the KSA.

## Institutional abbreviations

The collection abbreviations follow Evenhuis (2009) and Brandão (2000).

BMNH: The Natural History Museum, London, U.K.
KSMA: King Saud University Museum of Arthropods, Plant Protection Department, College of Food and Agriculture Sciences, King Saud University, Riyadh, Kingdom of Saudi Arabia.
MHNG: Muséum d’Histoire Naturelle, Geneva, Switzerland.
MNHN: Muséum National d’Histoire Naturelle, Paris, France.
NHMB: Naturhistorisches Museum, Basel, Switzerland.
WMLC: World Museum Liverpool, Liverpool, U.K.

## Measurements and indices Figs. 1–3:

All measurements are in millimeters and follow the standard measurements of previous works on the genus (Bolton, 1987; Sharaf et al., 2017):

EL: Eye Length; maximum diameter of eye in lateral view.
EM: Distance between anterior margin of eye and mandibular insertion in lateral view.
HL: Head Length; maximum length of head, excluding mandibles in full-face view.
HW: Head Width; maximum width of head behind eyes in full-face view.
ML: Mesosoma Length (=Weber Length); length of mesosoma in lateral view; from a point at which pronotum meets cervical shield to posterior base of propodeal lobes or teeth.
PPH: Postpetiole Height; maximum height measured in lateral view.
PPL: Postpetiole Length; maximum length measured in dorsal view.
PPW: Postpetiole Width; maximum width measured in dorsal view.
PTH: Petiole Height; maximum height measured in lateral view.
PTL: Petiole Length; maximum length measured in dorsal view, from anterior margin to posterior margin.
PTW: Petiole Width; maximum width measured in dorsal view.
PW: Pronotal Width; maximum width in dorsal view.
SL: Scape Length, excluding basal neck.
TL: Total Length, sum of lengths of head, mesosoma, petiole, postpetiole and gaster in profile.

## Indices

CI: Cephalic Index (HW/HL × 100).
EI: Eye Index (EL/HW × 100).
SI: Scape Index (SL/HW × 100).

## Additional Information and Declarations

### Data Availability

The following information was supplied regarding data availability: The raw data is included in the Results section.

### New Species Registration

The following information was supplied regarding the registration of a newly described species: Publication LSID:urn:lsid:zoobank.org:pub:58913E59-AEE4-43BD-881E-C23CD61F44F9; *Monomorium mohammedi* LSID:urn:lsid:zoobank.org:act:EA3405DE-CB2A-4E86-9511-2487920B7812.
